# Automated design of a convolutional neural network with multi-scale filters for cost-efficient seismic data classification

**DOI:** 10.1038/s41467-020-17123-6

**Published:** 2020-07-03

**Authors:** Zhi Geng, Yanfei Wang

**Affiliations:** 1 Key Laboratory of Petroleum Resources Research, Institute of Geology and Geophysics, Chinese Academy of Sciences, 100029 Beijing, P. R. China; 2 Innovation Academy for Earth Science, Chinese Academy of Sciences, 100029 Beijing, P. R. China; 3 University of Chinese Academy of Sciences, 100049 Beijing, P. R. China

**Keywords:** Geophysics, Seismology

## Abstract

Geoscientists mainly identify subsurface geologic features using exploration-derived seismic data. Classification or segmentation of 2D/3D seismic images commonly relies on conventional deep learning methods for image recognition. However, complex reflections of seismic waves tend to form high-dimensional and multi-scale signals, making traditional convolutional neural networks (CNNs) computationally costly. Here we propose a highly efficient and resource-saving CNN architecture (SeismicPatchNet) with topological modules and multi-scale-feature fusion units for classifying seismic data, which was discovered by an automated data-driven search strategy. The storage volume of the architecture parameters (0.73 M) is only ~2.7 MB, ~0.5% of the well-known VGG-16 architecture. SeismicPatchNet predicts nearly 18 times faster than ResNet-50 and shows an overwhelming advantage in identifying Bottom Simulating Reflection (BSR), an indicator of marine gas-hydrate resources. Saliency mapping demonstrated that our architecture captured key features well. These results suggest the prospect of end-to-end interpretation of multiple seismic datasets at extremely low computational cost.

## Abstract (web summary)

The authors present an automated design approach to propose a new neural network architecture for seismic data analysis. The new architecture classifies multiple seismic reflection datasets at extremely low computational cost compared with conventional architectures for image classification.

## Introduction

Applications of artificial neural networks (ANNs) are rapidly increasing in data-driven natural-science research fields such as materials^1–3^, biology and medicine^4–7^, and geoscience^8–10^. In exploration geophysics, many such studies can be treated as problems in visual image classification or segmentation. For example, geologists have used images of seismic reflection data to classify subsurface sedimentary units or hydrocarbon reservoirs^11^ and identify discontinuous structures like faults and large fractures^12^ or salt bodies^13^. All morphology patterns in such images can be properly learned by ANNs, many of which are based on the popular convolutional neural networks (CNNs) specifically designed for image-related tasks in computer vision. However, seismic reflection signals have intrinsically different natures compared with visual images with respect to polarity and limited bandwidth of sparse signals. In addition, seismic responses of geologic features vary in terms of wave propagation paths, frequencies, amplitudes, and polarity orientations. This suggests that data-driven ANN-based seismic interpretation research should be treated as complex mapping problems of high-dimensional sparse signals.

Morphology analysis of seismic images can employ CNNs for segmentation by pixel (e.g., fully convolutional network variants^12^) or classification by patch/image (e.g., Visual Geometry Group (VGG) or other variants^11,14^). In addition, CNNs used for classification have the potential to identify events or implications of multiple-channel seismic signals in a given receptive field that are difficult to annotate using pixel-level labels. For example, in geophysics and reflection seismology, wave amplitude variations by angle of incidence indicate types of hydrocarbons and drilling risk^15,16^. However, classic CNNs popular in computer vision classification are commonly manually designed by experience based on past designs and require enormous computational resources, even in recognition of 2D images. In addition, the seismic reflections of one specific subsurface reflector alone could consume gigabytes in storage space. Therefore, low-cost tools for handling high-dimensional seismic reflection signals are urgently needed. Specifically, such highly lightweight and computing efficient tools would significantly accelerate the estimation of marine seafloor hydrocarbon and methane hydrates resources, the carbon cycling of which has significant impacts on the atmosphere, biosphere, and hydrosphere from local to global scales^17–20^.

**Fig. 1:**
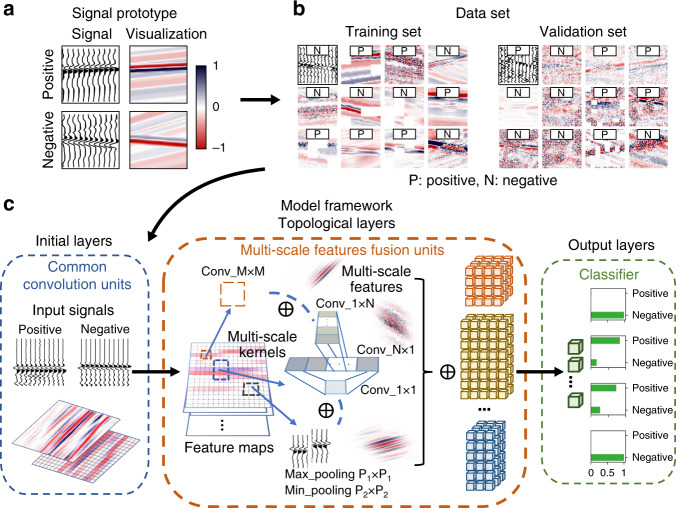
Schematic representation of data-driven CNN design flow proposed here. **a** Prototype of the target signal embedded in background signals. Key features differ in the combination of sequences of positive and negative “amplitudes”. Left-hand column presents wiggle plots of the signals and right-hand column presents 2D images of the corresponding signals. **b** Examples of heavily corrupted signals used for training and validation of the architecture candidates. **c** Diagram of the scalable architecture. The number and sizes of layers, kernels, and other components are variables to be determined by searching with respect to specific data and architecture space.

In this study, we propose a data-driven solution for automated searching a neural network architecture using a CNN framework capable of efficient seismic data classification (Fig. 1). We hypothesized that key signal features embedded in exploration seismic data could be captured by an ANN with significantly fewer parameters than classic CNN architectures. We first designed patches of conceptual signals (Fig. 1a) with particular sequences of seismic amplitudes; these are analogous to the key seismic reflections of oceanic gas hydrates. Next, various complex corruption methods were applied to the synthetic patches to generate an aggressive dataset (Fig. 1b) for searching the specific CNN architecture (Fig. 1c). The highlights in the CNN architecture were topological layers of fusion units used to filter multi-scale features. We factorized the network kernels to quadratically reduce the number of parameters and tried to keep polarity information by aggregating opposite sampling features. Our final architecture (SeismicPatchNet) was found by a random searching strategy with the help of high-performance graphics processing units (GPUs). To the best of our knowledge, this study constitutes the first data-driven design of a computationally efficient CNN intended for end-to-end interpretation of seismic data from the perspective of sparse-signal processing.

## Results

### Overview

For convenient comparative analysis, we focused on 2D data patches, meaning that the input tensor channel was limited to 1. We first presented the architecture evolution as a function of inference accuracy during massive searches of 167,512 instances in the architecture space and used this to detail the discovered SeismicPatchNet. We then benchmarked SeismicPatchNet along with several classic CNN architectures for image classification using our synthetic dataset. In addition, we evaluated these architectures’ predictive performance using real 3D seismic data from gas-hydrate exploration. Finally, we demonstrated that SeismicPatchNet could predict the bottom-simulating reflector (BSR) indicator for gas hydrates with: (1) the lowest number of architecture parameters and disk storage volume, (2) the largest inference speed and precision, and (3) high confidence for positive and negative features with minimum noise.

### Architectural evolution during search

**Fig. 2:**
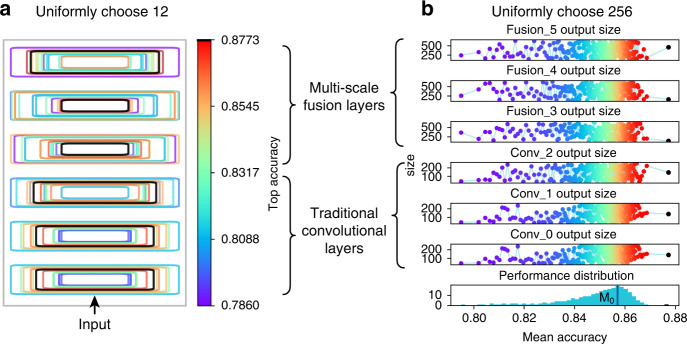
Evolution of network architectures. **a** Output size of network layers (rectangles). **b** Distribution of layer output size as a function of mean accuracy. Layers with matching color are from the same architecture; those with the best performance are shown in black. The mode (*M*_0_) of the distribution in **b** indicated the most frequently occurring accuracy of randomly drawn architectures. Relative sizes of rectangles in the group of traditional convolutional layers and multi-scale fusion layers were normalized separately.

A synthetic dataset was used to represent the worst-case seismic data scenario for massive searching of sub-optimal architecture in the architecture space. The searching task was distributed in multiple GPUs, which trained and validated 167,512 different architectures over the course of 1 month (Fig. 2a). As this was a problem of random searching instead of continuous optimization, for greater clarity, 12 instances were uniformly chosen from all architectures sorted by accuracy to show the evolution of CNN layers. We plotted the relative output size (rectangles in Fig. 2a) of the traditional convolutional layers and the topological fusion layers. The former were similar to the initial layers of GoogLeNet^21^ but with varying sizes; the traditional layers of the best architecture (black rectangles in Fig. 2a) were medium in size and the output size of each layer was similar. However, the latter exhibited a funnel form, in which the output size of the lower layer was significantly smaller than that of the higher layer. Similarly, we also uniformly chose 256 architectures to represent the overall performance, resulting in a negatively skewed distribution (Fig. 2b). The mode (*M*_0_) of the distribution in Fig. 2b indicated the most frequently occurring accuracy of randomly drawn architectures. Unexpectedly, the architecture performance at the mode was ~2% lower than at the optimal level, suggesting that the architecture with the best predictive performance was accidentally discovered and achieved a trade-off with layer output size.

### Configuration of SeismicPatchNet

The suggested configuration of SeismicPatchNet (Table 1) had seven layers/blocks containing trainable parameters in its naïve form. The size of input tensor was 112 × 112 × number of channels. The input receptive field covered a vertical area between 100 × 100 and 300 × 300 m^2^ in seismic surveys. The total number of trainable parameters was 0.725697 M. Overall, SeismicPatchNet had the following characteristics:

1. The output number of kernels/filters varied from layer to layer and was restricted to the specified searching space, preventing uncontrolled increases in feature maps and subsequent reduced computational performance.
2. The rich diversity of parallel multi-scale kernels/filters was concatenated as one output vector in each topological fusion layer, forming the input of the next layer (Fig. 1c). Similar to InceptionNet^22^, the factorization of convolutional operations was used to significantly reduce the number of trainable parameters: sequences of 1 × 1, *n* × 1, and 1 × *n* convolutions were combined to replace a single expensive *n* × *n* convolution with larger kernel size. In addition, a group of max-pooling and min-pooling operations was also incorporated as a parallel path to merge the sampled features from the previous layer. Our experimental experience suggested that adding pooling operations with opposite polarity had additional benefits on seismic reflection data.
3. In each topological layer, the size of kernels/filters and the number of various units were different, meaning that the fusion layers were not stacked upon each other with the same type as usual. All the numbers and *m* × *n* sizes of the kernels/filters were determined using an automated search strategy to approximate a quasi-optimal structure. Interestingly, both the number and size of the kernels showed a growing trend from the lower fusion layer (4) to the higher fusion layer (6), suggesting that patterns of higher abstraction were learned by higher layers^21^.
4. Only traditional and most used operations like convolution and feature sampling/pooling were considered, in order to significantly reduce the computational resources needed and thus improve computing speed; more intermediate variables during the training mean more memory usage and less computational efficiency. In the naïve form of SeismicPatchNet, all layers used rectified linear unit as activation function. Occasional pooling layers with stride two were used to halve the resolution of the feature maps. One dropout layer followed by one fully connected layer was applied to combat the overfitting problem.

**Table 1:** Configuration of SeismicPatchNet.

Type (layer lb.)	Patch size/stride	Output size	Element (size): num.
Convolution (1)	7 × 7/2	56 × 56 × 137	Conv (7 × 7): 137
Max pool	3 × 3/2	28 × 28 × 137	Max pool (3 × 3): 1
Convolution (2)	1 × 1/1	28 × 28 × 137	Conv (1 × 1): 137
Convolution (3)	3 × 3/1	28 × 28 × 144	Conv (3 × 3): 144
Max pool	3 × 3/2	14 × 14 × 144	Max pool (3 × 3): 1
Fusion (4)	NA^a^	14 × 14 × 133	Conv (2 × 2): 83
			Conv (1 × 1): 104
			Conv (3 × 1): 2, conv (1 × 3): 2
			Conv (1 × 1): 112
			Conv (7 × 1): 16, conv (1 × 7): 16
			Max pool (2 × 2): 12, min pool (2 × 2): 11
			Max pool (3 × 3): 4, min pool (3 × 3): 5
Fusion (5)	NA^a^	14 × 14 × 151	Conv (2 × 2): 75
			Conv (1 × 1): 280
			Conv (5 × 1): 5, conv (1 × 5): 5
			Conv (1 × 1): 382
			Conv (7 × 1): 7, conv (1 × 7): 7
			Max pool (2 × 2): 20, min pool (2 × 2): 21
			Max pool (5 × 5): 12, min pool (5 × 5): 11
Max pool	3×3/2	7 × 7 × 151	Max pool (3 × 3): 1
Fusion (6)	NA^a^	7 × 7 × 459	Conv (2 × 2): 14
			Conv (1 × 1): 46
			Conv (3 × 1): 25, conv (1 × 3): 25
			Conv (1 × 1): 170
			Conv (5 × 1): 116, conv (1 × 5): 116
			Max pool (4 × 4): 107, min pool (4 × 4): 107
			Max pool (5 × 5): 45, min pool (5 × 5): 45
Average pool	7×7/1	1 × 1 × 459	Average pool (7 × 7): 1
Dropout (50%)	NA^a^	1 × 1 × 459	NA^a^
Linear_FC (7)	NA^a^	1 × 1 × 459	Fully connected
Classifier	NA^a^	1 × 1 × 64	Softmax

^a^*NA* not applicable here.

### Computational performance using the synthetic dataset

**Fig. 3:**
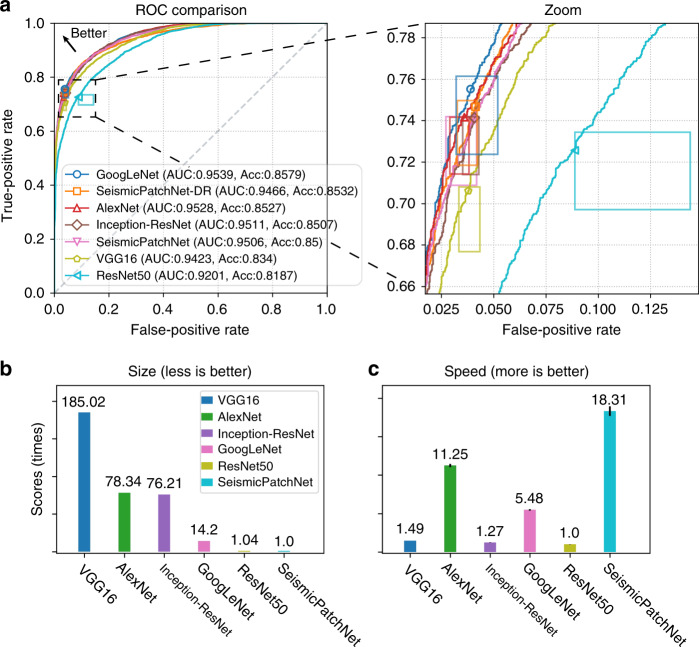
Prediction performance of typical CNN architectures using synthetic test data. **a** Receiver operator characteristic (ROC) curves with corresponding zoomed-in plot. Markers with matching color are the position of the 0.5 confidence threshold of the best-trained model, while the corresponding rectangle in the zoomed-in plot denotes variations of the threshold during dozens of training runs. Normalized comparison of architectures for **b** parameters size and **c** inference speed. SeismicPatchNet-DR SeismicPatchNet trained with a double regularization method, AUC area under the ROC curve, Acc predictive accuracy on the test dataset. The ResNet50 architecture used was a realization for the CIFAR-10 dataset^24^.

Receiver operator characteristic (ROC) curves (Fig. 3a) were used to assess the comprehensive performance of SeismicPatchNet and five other classic CNN architectures using the synthetic test dataset. All CNN architectures were trained dozens of times using similar settings, including moving averages of the trainable parameters/weights and automated training, to allow accurate statistical comparison of their performance. Markers on each ROC curve in Fig. 3 indicate the position of the 0.5 confidence threshold of the best-trained model, while the corresponding rectangle denotes the variations of the threshold during the dozens of training runs. Unexpectedly, ResNet-50^23^, which has shown superior performance over other classic CNN architectures in visual image recognition tests^24^, performed worst on our synthetic seismic data. The prediction accuracy and the area under curve (AUC) value for ResNet50 were ~4% and ~3% less than that of GoogLeNet^21^, respectively, which had the best performance. In addition, the performance of VGG-16^25^, which contained the largest number of parameters, was moderate compared with all others except ResNet-50. The overall performance of SeismicPatchNet and its model trained with a double regularization method was similar to the other CNN architectures. The performance of Inception-ResNet^22^, which combines GoogLeNet variants and ResNet, fell between that of GoogLeNet and ResNet-50. In summary, the architectures with feature fusion designs (GoogLeNet and SeismicPatchNet) were overall superior to the others, especially those with skip connections^24^ (e.g., ResNet).

However, there were remarkable differences among the CNN architectures with regard to the number of parameters (size in Fig. 3b) and computing speed (Fig. 3c). In this study, VGG-16 consisted of 16 layers with ~134 M trainable parameters and took up nearly 500 MB of storage space, 185.02 times larger than SeismicPatchNet. In contrast, the best predictive accuracy and AUC for SeismicPatchNet was 1.9% and 0.43% larger than for VGG-16, respectively. Although GoogLeNet performed slightly better (Fig. 3a), its size was ~14 times that of SeismicPatchNet (Fig. 3b). On the other hand, the computing speed of SeismicPatchNet was more than 12 and 18 times higher than that of VGG-16 and ResNet-50, respectively. SeismicPatchNet had a comparable number of trainable parameters with ResNet-50 but had an order-of-magnitude advantage in both low memory usage and high predictive speed. Using the same settings, ResNet-50 could only be trained using a large-memory GPU (24 GB, Titan RTX Nvidia GPU) because of expensive operations in the architecture such as the batch normalization of parameters. In most cases, automated training of ResNet-50 took ~2 h that of SeismicPatchNet took only ~8 min. Thus, SeismicPatchNet clearly outperformed other CNN architectures in a combination of computational cost and predictive speed.

### Comparison of predictive performance using real data

**Fig. 4:**
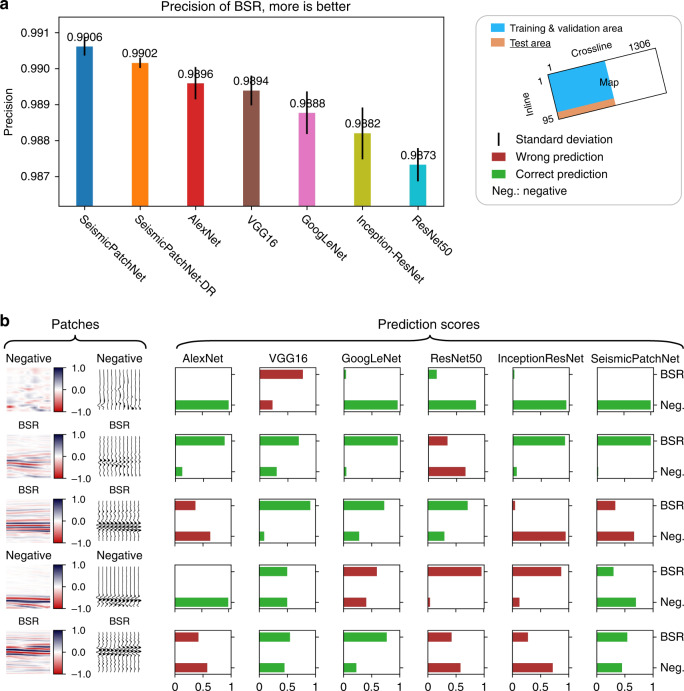
Predictive performance of CNN architectures using gas-hydrate field data from Blake Ridge, USA. **a** Comparison of predictive precision using real seismic data from the test area showed in the legend. **b** Selected illustrations of the inferred occurrence of BSR by the CNN architectures. SeismicPatchNet-DR SeismicPatchNet trained with double regularization method. The occurrence of BSR is represented by probability (0–1).

3D seismic reflection data of oceanic gas hydrates from Blake Ridge (USA)^26,27^ were used to evaluate the real-world performance of the various CNN architectures (Fig. 4). All architectures were trained five times using a similar procedure and a trained model representing the average performance of each was chosen for further comparison. As BSR was the only prediction focus, precision was used to assess the predictive performance (Fig. 4b).

SeismicPatchNet clearly achieved higher precision for real-world BSR prediction than the other CNN architectures (Fig. 4a), though its advantage was only 0.33% greater than the lowest performer (ResNet-50, as in the first test). Interestingly, GoogLeNet’s performance using real-world data was lower than for synthetic data; as this contained one order of magnitude more parameters than SeismicPatchNet, it may have been inferior at capturing the sparse features of real seismic reflection signals. In addition, although SeismicPatchNet was marginally superior to the architecture trained with double regularization (SPN-DR), the latter had less performance variation. As real-world seismic reflection signals are particularly sparse, SeismicPatchNet inheriting implicit regularization ability may be capable of covering the data space even without the use of explicit regularization schemes to improve the network’s generalization performance.

### Attentive responses of the CNN architectures

**Fig. 5:**
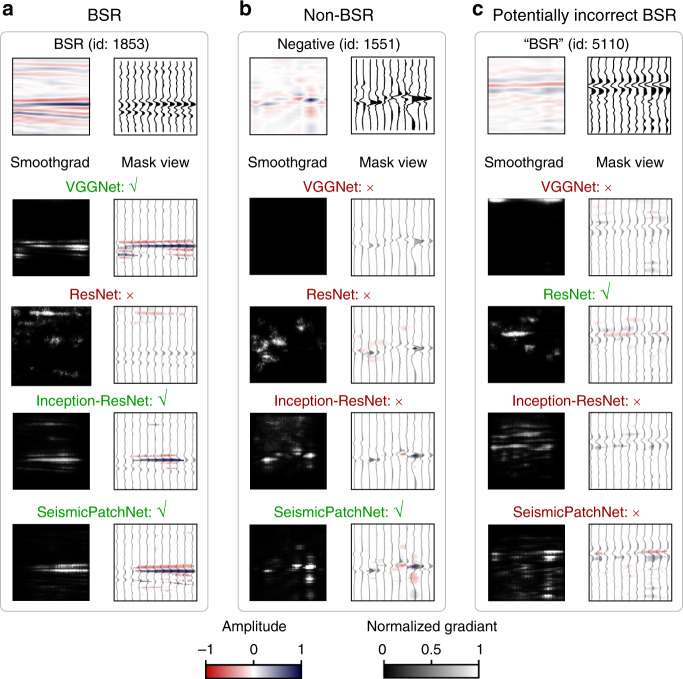
Selected examples of attentive responses by representative CNN architectures to real-world data. **a** Ground-truth BSR seismic patch. **b** Ground-truth non-BSR seismic patch. **c** Seismic patch potentially incorrectly labeled as BSR. The activation of CNNs (guided-smooth-gradient) and the masked map are represented by grayscale images (left column) and superimposed signal plots (right column), respectively. Only the labels were used to judge the correctness.

We performed attentive response analysis using a guided-smooth-gradient algorithm^28^ to identify the prediction focus of the CNN architectures (see more examples of SeismicPatchNet in Supplementary Fig. 3) with respect to label class. The key signal features used by a specific CNN-trained model for prediction were determined by superimposing maps of the masked tensor of the salient gradient and the signals with polarity. For demonstration purposes, we selected three patches from the real seismic dataset, including a ground truth BSR (Fig. 5a), a ground truth non-BSR (Fig. 5b), and a potentially incorrectly labeled BSR (Fig. 5c). The key feature in a BSR patch is a sequence of a negative signal followed by a positive signal. In Fig. 5a, all architectures except ResNet-50 properly captured this pattern. However, in Fig. 5b, only SeismicPatchNet was sensitive to both strong reflections and background features. Although Inception-ResNet showed a salient gradient (grayscale image in Fig. 5b) around the strong reflections, it mainly emphasized the positive signals. In Fig. 5c, SeismicPatchNet once again showed that it was sensitive to both strong reflections and a few background features. More examples (Supplementary Fig. 3) also demonstrated that SeismicPatchNet could learn patterns that agreed with accepted seismic reflection signatures.

### Predictive performance of CNNs in field applications

**Fig. 6:**
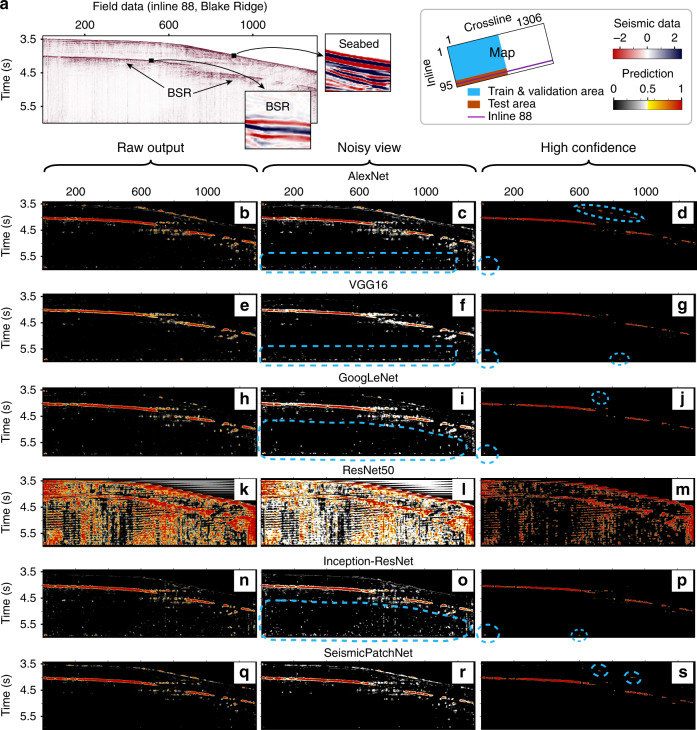
Predictions on a section (inline 88) of 3D seismic data from the Blake Ridge gas hydrates. **a** Image view of the seismic data (inline 88). **b**–**s** show BSR occurrence predicted by selected CNN architectures. Raw output: the original output of the trained models; the output data are provided as source data. Noisy view: the predicted value belonging to [0.05, 0.95] is set to 0.5. High confidence: the predicted value belonging to [0, 0.95] is set to 0.

A complete section of the 3D seismic data (inline 88, Fig. 6a) was chosen to test the predictive performance of the CNN architectures for characterizing the subsurface distribution of BSR (Fig. 6b–s). Unexpectedly, ResNet-50 failed to characterize the occurrence of BSR in the field using the same prediction method as the others. In the noisy view of the predictions (the middle group of plots in Fig. 6), there was also much more noise (white dots) in the deep zone results (dashed line in the middle group) of Inception-ResNet (Fig. 6o) than in the results of SeismicPatchNet (Fig. 6r), which showed the highest confidence for non-BSR. However, all the architectures showed false positives of BSR more or less along the seabed, except VGG-16. As shown by the zoomed-in seabed image in Fig. 6a, the leading polarity of the seabed reflection was very similar to that of BSR, probably because of technical issues in the data processing. This means that the seabed reflection signatures around the marked region were misleading and inconsistent with the accepted definition, explaining the false positives of CNN architectures including SeismicPatchNet. For comparison, only SeismicPatchNet showed high confidence in non-BSR in the deep zone (the right group of plots in Fig. 6). In practice, however, the false positives along the seabed can be easily removed by computational post-processing, but those in the subsurface zones cannot be removed without human intervention. Therefore, this case study demonstrated the robustness of SeismicPatchNet in this application.

## Discussion

Neural architecture search (NAS) has been one of the most popular topics in machine learning in recent years, with many studies focusing on search strategies like reinforcement learning, evolutionary algorithms, and Bayesian optimization^29^. One common issue, however, is that most NAS research has placed a priority on inference performance and consequently tends to produce a large architecture^30^. Here, we used a random search^31^ strategy and hypothesized that a significantly small architecture could be found if the draw number was large enough. Although random search is believed to be computationally expensive, it is a global optimization method for hyper-parameter selection in deep learning, and other research has shown that the resultant architectures were comparable to those found by other optimization processes in a certain period of computation time^30–32^. By limiting the number of topological fusion layers with rich multi-scale filters used to aggregate complex features of the polar signals, we demonstrated a trade-off between search efficiency and architecture performance. As it is impossible to draw infinite realizations from the search space, our core intention was the accidental discovery of an architecture with significantly few parameters (Fig. 2b and Table 1), which may not be acquired by some adaptive/optimization algorithms trapped at local convergences.

We therefore proposed a data-driven solution to search a compact CNN architecture (SeismicPatchNet) devoted to classifying multiple seismic reflection datasets synchronously at a low computational cost. After constructing a complex but quantitatively controllable data space to simulate seismic data in a difficult scenario, a sub-optimal architecture for sparse signals was found through hundreds of thousands of automated searches in a restricted architecture space. Unlike the usual practice of stacking the same layers repeatedly, we designed topological fusion layers with rich multi-scale filters. For comparison, the traditional convolutional layers and fully connected layer used in classic CNNs were kept in SeismicPatchNet to show the advantages of the newly designed topological fusion modules. Only some regular operations like traditional convolution, activation, and sampling were employed for balancing the computational resources. Many other cutting-edge operations, such as dilated convolutions^33^ and transposed convolutions^34^, may contribute to the overall inference performance of the architecture; these need further investigate in future research. However, the poor performance of ResNet50 using the synthetic data and the noisy outputs of Inception-ResNet and ResNet50 on the real data suggested that skip connections^24^ deteriorated the architectures’ inference performance on our seismic data. A potential explanation was that the effective features in polarized seismic data were sparse and different from vision images, such that the skip connections passed useless information (noises) from shallow to deep layers. On the other hand, total number of parameters of the cutting-edge architectures were commonly in the dozens of millions, but only 0.73 million in our architecture. Although the functional capacities of those architectures should therefore be nearly 100 times greater than our architecture, their performances were far from superior to ours. Therefore, we concluded that our architecture had comparable inference performance and achieved a major advancement in computational speed and resource efficiency.

We demonstrated that the naïve form of SeismicPatchNet was equal or better, in terms of predictive performance, than some state-of-the-art CNN architectures when used on both synthetic and real-world seismic data. Although we used stacked seismic data with one channel, SeismicPatchNet showed order-of-magnitude superiority in computing speed with a significantly smaller number of trainable parameters. Thus, variants of SeismicPatchNet can be used to process multiple channels of seismic reflection signals directly deployed on individual devices with limited computational resources. This should make it possible to conduct efficient end-to-end interpretations of subsurface geological implications, which are not problems of visual image recognition. For example, under certain condition, VGG-16 would consume ~55 GB of GPU memory to train using a partial-stack seismic dataset with multiple channels (offsets), while SeismicPatchNet only needed ~300 MB memory. Specifically, smaller and more powerful variants of the architecture could be proposed by carefully optimizing the shallow network of traditional convolutional layers, considering a scale-free topology^35^, or replacing the fully connected hidden layer with sparse ones^35^. We hope that these results will stimulate new research into automated machine learning for seismic data interpretation, and help explore marine carbon resources indicated by seismic data at a decisive advantage in terms of computational efficiency and cost.

## Methods

### Synthetic data

To develop a data space that was analogous to exploration-derived seismic data and could be quantitatively controlled, we designed a synthetic dataset by embedding stochastic key signals in chaotic background signals. The original data prototypes were patches consisting of stochastic sequences of bricks with different thicknesses and values ranging from −1 to 1 with a mean of 0 (Fig. 1a), imitating varying-amplitude reflections of stratigraphic units. The key signal in the patch labeled as True (coded as 1) was a combination of one negative amplitude (−1 to 0) followed by one positive amplitude (0 to 1). The key signal in the patch labeled as False (coded as 0) was either a combination of one positive amplitude followed by one negative amplitude or nothing but fully random background signals. The patches with True labels were analogous to BSR, seismic reflections indicating the base of oceanic gas hydrates^36^.

In contrast, the key features of the seabed reflections were a reverse combination of the wave polarity (one positive amplitude followed by one negative amplitude). The synthetic patches represented seismic reflections for BSR, non-BSR, and seabed/environmental/other settings. To push the limit of the predictive performance of our CNN architectures, we applied a series of corruption methods when processing the patch data, including random brightness, blur, Gaussian noise, elastic transformation, frequency noise, perspective transformation, and coarse dropout, then prepared the synthetic dataset for testing. The synthetic data were particularly designed to simulate extreme situations in the seismic reflection data, such as significant low signal-to-noise ratio or brightness, serious noise problems, and missing records.

We used 16,000 patches (samples) for training and 4000 patches for validation during massive searching of the CNN architecture. A more complex dataset containing 7500 patches was used as benchmark data when comparing CNN performance. All data were approximately balanced, and only the patches containing BSR were labeled as True. All synthetic data were generated by the same pipeline but with different controlling parameters to differentiate the data space quantitatively while assessing the architectures’ capability to focus on key signals. Randomly selected patches of the training set and the synthetic benchmark data are shown in Supplementary Fig. 1. More information about the synthetic data and patch generation and processing are detailed in the open-source codes.

### Real-world seismic data

The real-world data consisted of 3D marine exploration seismic data from the well-studied Blake Ridge gas-hydrate site offshore of South Carolina, USA. The seismic survey covered an area of 348.93 km^2^ with two-way time ranging from 3.400 to 5.998 s. The crossline 1 to 690 of inline 6 to 80 was chosen for preparing the training and validation dataset, while that of inline 81 to 92 was chosen for preparing the test data (Fig. 4).

Unlike those for fault morphology, BSR reflections have complex regional features because of their complicated geologic origin^37^. We manually annotated BSR occurrences in seismic data qualified in previous research^26,38^. Before producing the labeled dataset for machine learning, the seismic data were normalized to between −1 and 1. Four types of patches were extracted by sliding windows at a step increment of 5 traces along with the BSR locations, BSR vicinity zones, seabed, and other zones. The patch window covered 140 ms of depth in a two-way time domain and 7 traces with spacing of 37.5 m. Only the patches contained BSR were labeled as True and the data represented an approximately balanced dataset. No data augmentation methods were applied except flipping the patches horizontally. A total of 30,478 patches were used to train the CNN architectures (25,905 for training, 4,573 for validation) and 5,339 patches were used as test data for the benchmark. Randomly selected patches of the training set and the benchmark real-world data are shown in Supplementary Fig. 2.

### Architecture search and training procedure

To limit the architectures’ space, we defined a template containing three traditional convolutional layers (initial layers in Fig. 1c), three topological layers/blocks with multi-scale fusion units (topological layers in Fig. 1c), and one fully connected hidden layer for controlling output size (output layers in Fig. 1c), ending with one Softmax classifier: $P\left( {y = j|x} \right) = \frac{{e^{x^Tw_j}}}{{\mathop {\sum}\nolimits_{k = 1}^K {e^{x^Tw_k}} }}$. All the sizes of the layers’ output, the multiple kernels/filters inside the layers (Fig. 1c), and the configuration of kernels/filters are discrete integer variables belonging to certain intervals. The output sizes of the traditional convolutional layers belonged to [32, 256], while the output sizes of the topological layers/blocks belonged to [128, 640]. The sum of numbers of the kernels/filters inside the corresponding layer equaled the layer’s output size. The size of kernels/filters in the layers was determined as $conv_ - M \times M:\,M \in \left[ {1,2} \right]$, $conv_ - 1 \times N:N \in \left[ {3,\,7} \right]$,$pooling\,size \in \left[ {2,\,5} \right]$ (Fig. 1c). Consequently, hundreds of thousands of CNN architectures with the same topological framework but with different functional capacities were randomly generated, trained, and validated during massive searches carried out by high-performance GPUs. Then, we sorted the trained models by inference accuracy with regard to the validation data. Finally, the architecture corresponding to the trained model with the highest accuracy and a significantly small number of parameters was selected as the desired architecture. The pseudocode of the architecture search strategy was summarized in Table 2.

**Table 2:** Architecture search strategy.

Algorithm 1: random search pseudocode
(1) Build a complex data space *χ* and architecture configuration space *λ*;
(2) Define a learning/training algorithm ${\Bbb A}$ for mapping dataset *χ*^train^ to a function $f = {\Bbb A}_\lambda \left( {w,\chi ^{train}} \right)$ as a trained model;
(3) **while** not stopped **do**
(4) Randomly draw trial points $\left\{ {\lambda ^1 \cdots \lambda ^n} \right\}$ in *λ* to create network realizations;
(5) Update weights/parameters *w* of networks by descending loss ${\cal{L}}\left( {x;f} \right)$;
(6) Log performance evaluations;
(7) Derive the final architecture with high performance and small number of parameters.

The training and prediction algorithms were implemented using Python and TensorFlow^39^. For a fair comparison, all CNN architectures were trained and evaluated using the same procedure and similar settings. We adopted an automated training and stopping strategy to guarantee full training without overfitting. In this approach, the program would not stop training if the loss value on the validation data decreased more than 2.5% relative to the minimum loss value within a certain number of iteration steps, during which ~70% of the whole training dataset was consumed. We used the Adam optimizer^40^ and exponential moving average method^39^ to update the trainable parameters. As the number of training epochs varied significantly in different architectures, we set a constant learning rate of 10^−4^ for the optimizer. The training was performed with mini-batches of 64 patches for each epoch and cross-entropy $( {C = - \mathop {\sum}\nolimits_{c = 1}^M {y_{o,c} \cdot log( {p_{o,c}} )} } )$ as the loss function. The massive searching of architectures took ~1 month in machines equipped with one Titan RTX and three RTX 2080ti Nvidia GPUs.

### Hybrid regularization scheme

Inversion problems in seismic reflection benefit from hybrid regularization methods^41^ due to the sparsity and non-smoothness of seismic data. Inspired by this, we adopted the following regularization scheme to train and explore the performance limit of our architecture using an extreme seismic data scenario:

1 $$J^{\alpha ,\beta }\left( W \right) = - \mathop {\sum}\limits_{c = 1}^M {y_{o,c} \cdot {\mathrm{log}}( {p_{o,c}} ) + \alpha \left\| W \right\|_{l_1} + \beta \left\| W \right\|_{{\mathrm{TV}}}}$$

where *J* is the objective function to be minimized by the optimizer, *W* is all the trainable parameters, $- \mathop {\sum}\nolimits_{c = 1}^M {y_{o,c} \cdot {\mathrm{log}}( {p_{o,c}})}$ is the cross-entropy loss function, *M* is the number of categories/classes, *y*_*o,c*_ is the indicator/label (0 or 1), *p*_*o,c*_ is the prediction score that the observation sample *o* belongs to category *c*, and *α* and *β* are penalty parameters for the *l*_1_ and TV regularization of *W*, respectively. The term $\alpha \left\| W \right\|_{l_1}$ is used to keep the sparsity of the trained model’s parameters, while the term $\beta \left\| W \right\|_{TV}$ is used to keep the boundary structure/smoothness of the parameter space. The double regularization scheme was implemented in TensorFlow. The penalty parameters were tuned by random searching of a given parameter space.

### Benchmark and robustness of the CNNs

All the trained models of CNN architectures were restored with the moving-averaged parameters for evaluation and prediction using the test data, contributing to the robustness of the training and prediction performance of the individual architecture. As we were limited by the computational resources and experimental time available, we also trained the CNN architectures on the synthetic dataset for dozens of time to compare the statistical performance. Because of the simplicity of the real seismic data, the CNN architectures were trained five times for each on the real dataset. We also monitored training curves of validation loss and accuracy for quality control. The ResNet50 used was a realization for the CIFAR-10 dataset^24^. The computational speed and the number of parameters of the CNN architectures were normalized for comparison; the results may differ by software library version and hardware specifications.

### Prediction using real-world data

**Fig. 7:**
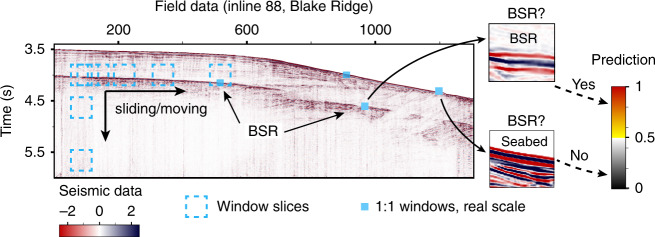
Predicting of BSRs on a seismic data section using a simple sliding window method. The dotted rectangles are illustrations of slice windows moving row by row on specific seismic section, while the solid squares denote the windows with real scale on the section. The prediction results of BSR occurrence is represented by probability (0 to 1).

We predicted BSR occurrence by feeding the CNN architectures with patches of real seismic data extracted from 2D sections of the 3D data cube using the sliding window approach^42^ (Fig. 7). These patches were sliced by a fixed-size window from the top-left corner to the bottom-right corner of the 2D seismic section (inline 88), at a moving step of 5 seismic traces in the lateral direction and 6 ms of two-way time in depth. Prediction scores given by the CNN architectures were plotted as a prediction confidence map (Fig. 6), ranging from 0 (definite non-BSR) to 1 (definite BSR).

### Interpretable analysis of salient features

Interpretable studies highlight the relevant focus of the CNN architectures on the object of interest. We employed a guided-smooth-gradient algorithm^28^, implemented in the Python repository (PyPI), to show the responses of the trained model’s parameters with respect to the pointwise intensities of the input seismic signals (Fig. 5). The smoothed gradients were converted to 2D grayscale images, the brightness of which indicated the attention of the architecture. To visualize the polarity of the seismic signals, we also plotted the wiggle view of seismic traces overlapped by the masked image of the seismic patch. Only 20% of the most salient pixels were shown. The opacity of the seismic traces and the image were set to 30 and 95%, respectively, for improved visual perception.

## Supplementary information

Supplementary Information

## Data Availability

The synthetic data for architecture searching were generated using the scripts in the code repository mentioned below. The real seismic data of Project Blake Ridge Hydrates is available under a Creative Commons (CC BY-SA) license at http://www.opendtect.org/osr/Main/BlakeHydrates. The source data underlying Figs. 6b, e, h, k, n and q (raw output of trained models) are provided as a Source Data file. All other relevant data are available upon request.

## Source data

Source Data

## Code availability

Code supporting the findings of this study are available open-source in GitHub.
