# Author Correction: Automated design of a convolutional neural network with multi-scale filters for cost-efficient seismic data classification

**DOI:** 10.1038/s41467-020-19282-y

**Published:** 2020-10-22

**Authors:** Zhi Geng, Yanfei Wang

**Affiliations:** 1 Key Laboratory of Petroleum Resources Research, Institute of Geology and Geophysics, Chinese Academy of Sciences, 100029 Beijing, P. R. China; 2 Innovation Academy for Earth Science, Chinese Academy of Sciences, 100029 Beijing, P. R. China; 3 University of Chinese Academy of Sciences, 100049 Beijing, P. R. China

**Keywords:** Geophysics, Seismology

Correction to: *Nature Communications* 10.1038/s41467-020-17123-6, published online 3 July 2020.

The original version of this Article contained a typo in Eq. (1), in which the subscript of $\beta \left\| W \right\|_{{\mathrm{TV}}}$ was given incorrectly. The correct expression is $\beta \left\| W \right\|_{l_2}$. Correspondingly, in the following text, “TV regularization of *W*” should be corrected as “*l*_2_ regularization of *W*”; “$\beta \left\| W \right\|_{{\mathrm{TV}}}$ is used” should be corrected as “$\beta \left\| W \right\|_{l_2}$ is used”.

