# Supplementary material for: Automated design of a convolutional neural network with multi-scale filters for cost-efficient seismic data classification
Source: Nat Commun. 2020 Jul 3;11:3311. doi: 10.1038/s41467-020-17123-6 (PMC7335201; doi:10.1038/s41467-020-17123-6)
Supplement: Supplementary file 1 — Supplementary Information [file 41467_2020_17123_MOESM1_ESM.pdf]

Supplementary Information for *Nature Communications*

Automated design of a convolutional neural network with multi-scale filters for cost-efficient seismic data classification

by Geng et al.

Supplementary Figures

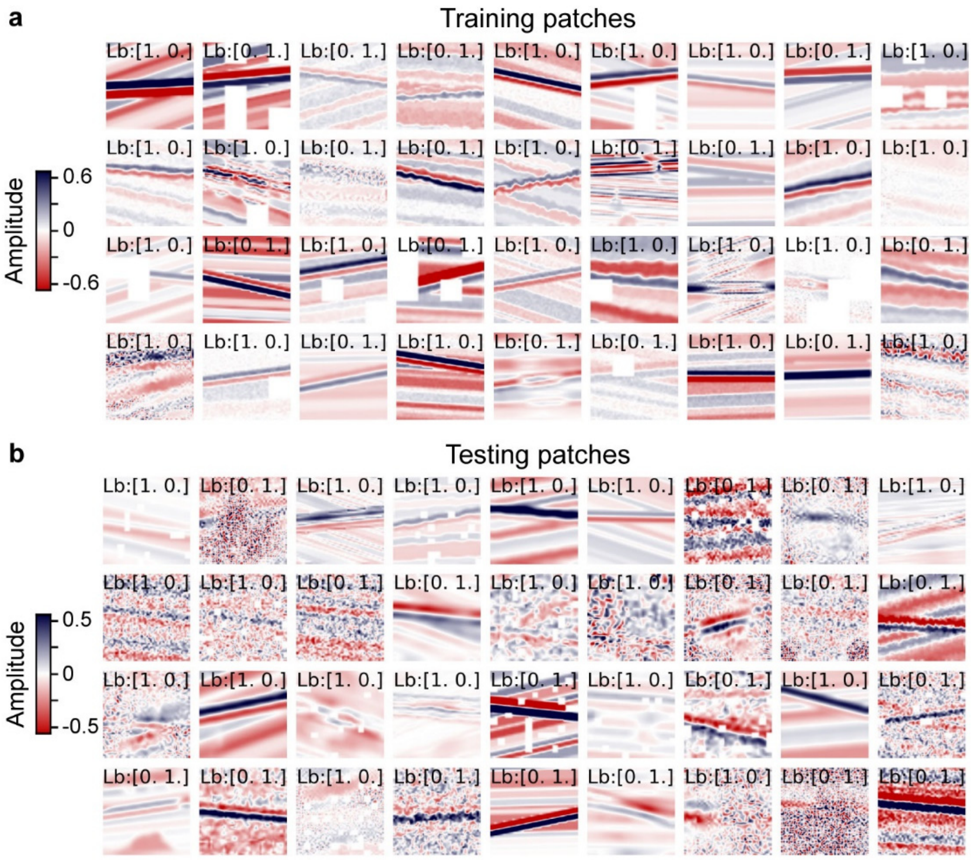

Supplementary Fig. 1 Randomly selected images of the synthetic data patches for **a** training and **b** testing. The synthetic test data were generated by a more complex data space than that used to generate the training and validation data. Patches with labels [0, 1] are seismic reflections of BSR, while those with labels [1, 0] are seismic reflections of non-BSR.

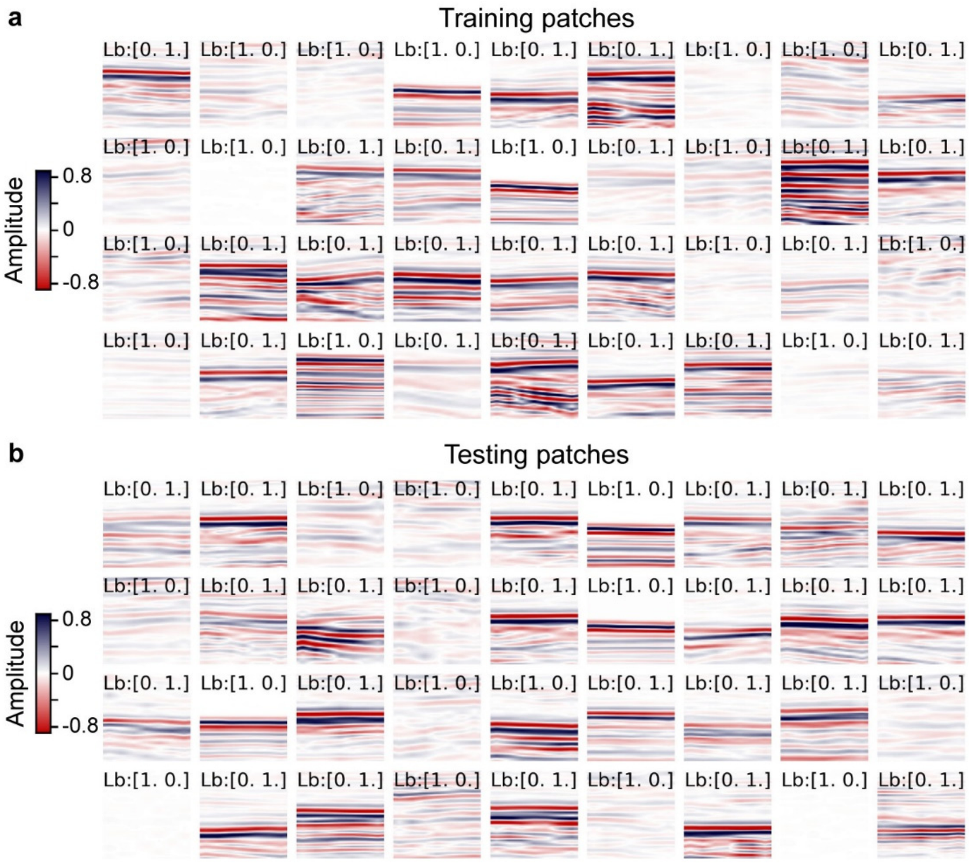

Supplementary Fig. 2 Randomly selected images of the real data patches for **a** training and **b** testing. Patches with labels [0, 1] are seismic reflections of BSR, while those with labels [1, 0] are seismic reflections of non-BSR.

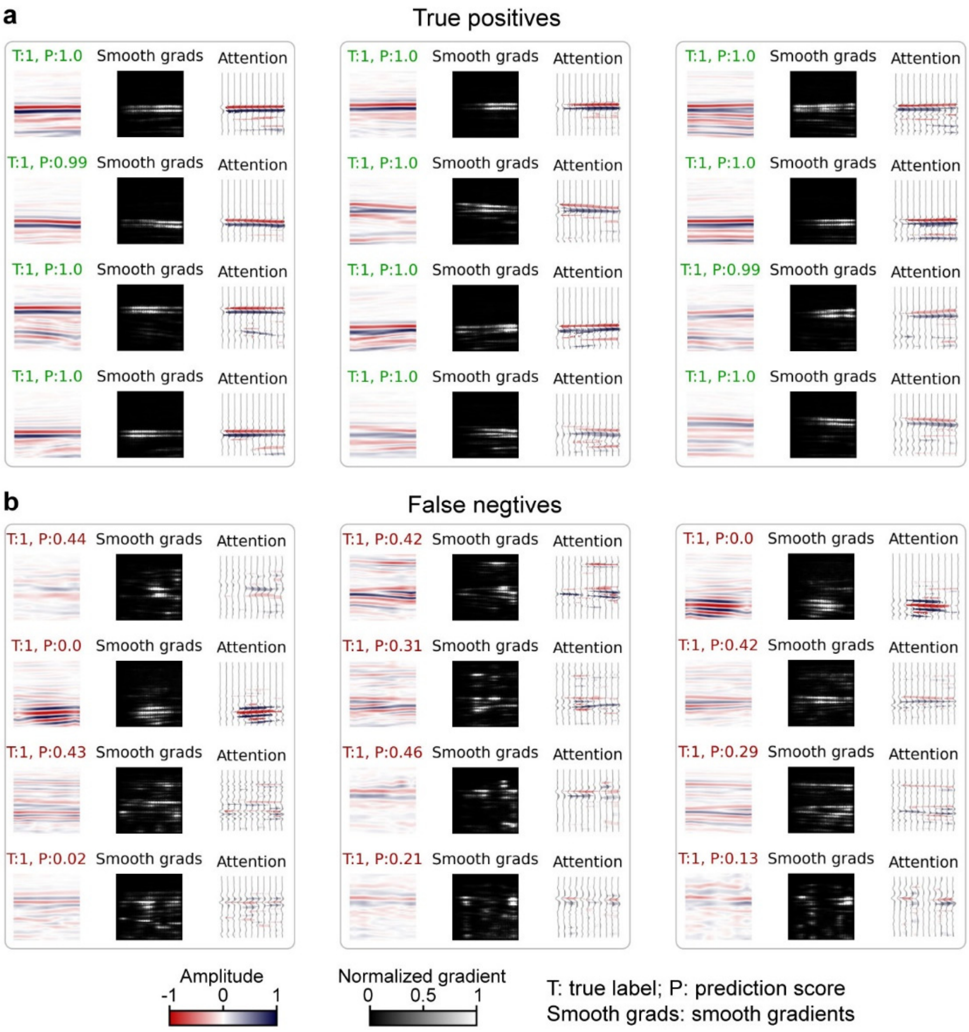

Supplementary Fig. 3 Selected examples of attentive responses analysis for SeismicPatchNet. a Attention maps of true positives, suggesting that SeismicPatchNet mainly focused on the key polar signals of BSR. **b** Attention maps of false negatives, suggesting that SeismicPatchNet paid attention to features of both the BSR and the background.
